# Adolescent but not adult ethanol binge drinking modulates cocaine withdrawal symptoms in mice

**DOI:** 10.1371/journal.pone.0172956

**Published:** 2017-03-14

**Authors:** Juan Carlos Ledesma, Maria A. Aguilar, Pablo Giménez-Gómez, José Miñarro, Marta Rodríguez-Arias

**Affiliations:** Departament de Psicobiologia, Universitat de València, Avda. Blasco Ibáñez 21, Valencia, Spain; Universidade do Estado do Rio de Janeiro, BRAZIL

## Abstract

**Background:**

Ethanol (EtOH) binge drinking is an increasingly common behavior among teenagers that induces long-lasting neurobehavioral alterations in adulthood. An early history of EtOH abuse during adolescence is highly correlated with cocaine addiction in adulthood. Abstinence of cocaine abuse can cause psychiatric symptoms, such as anxiety, psychosis, depression, and cognitive impairments. This study assessed the consequences of adolescent exposure to EtOH on the behavioral alterations promoted by cocaine withdrawal in adulthood.

**Methods:**

We pretreated juvenile (34–47 days old) or adult (68–81 days old) mice with EtOH (1.25 g/kg) following a binge-drinking pattern. Then, after a three-week period without drug delivery, they were subjected to a chronic cocaine treatment in adulthood and tested under cocaine withdrawal by the ensuing paradigms: open field, elevated plus maze, prepulse inhibition, tail suspension test, and object recognition. Another set of mice were treated with the same EtOH binge-drinking procedure during adolescence and were tested immediately afterwards under the same behavioral paradigms.

**Results:**

Adolescent EtOH pretreatment undermined the anxiogenic effects observed after cocaine abstinence, reduced prepulse inhibition, and increased immobility scores in the tail suspension test following cocaine withdrawal. Moreover, the memory deficits evoked by these substances when given separately were enhanced in cocaine-withdrawn mice exposed to EtOH during adolescence. EtOH binge drinking during adolescence also induced anxiety, depressive symptoms, and memory impairments when measured immediately afterwards. In contrast, neither EtOH nor cocaine alone or in combination altered any of these behaviors when given in adulthood.

**Conclusions:**

EtOH binge drinking induces short- and long-term behavioral alterations and modulates cocaine withdrawal symptoms when given in adolescent mice.

## 1. Introduction

Many studies indicate that ethyl alcohol (ethanol, EtOH) is one of the first drugs of choice among young people, and that binge drinking is increasingly frequent among this age group in numerous countries [1–3]. A “binge” is a pattern of EtOH intake that results in the blood's alcohol concentration reaching 0.08-gram percent or above; i.e., five or more drinks consumed over the same period of time [2]. Concerns about heavy drinking during adolescence, which is a critical stage of brain maturation, have grown since recent evidence has demonstrated that it can have a negative impact on brain structure and function, causing significant short- and long-term neurobehavioral disturbances [4–7]

Interestingly, it has been reported that early onset and higher frequency of EtOH consumption is a reliable predictor of later problematic abuse of other illicit drugs in adulthood [8–9] This is known as the “gateway hypothesis”, which states that a causal relationship exists between early exposure to drugs and the abuse of other substances later in life [10–12]. In accordance with this theory, it has been demonstrated that addiction to cocaine, the most used illicit drug in developed countries after cannabinoids, is strongly correlated with an early history of EtOH abuse in adolescence [13–15]. Cocaine addiction is a chronic brain illness characterized by compulsive intake and relapse after short periods of abstinence [16]. Abuse of this substance and its subsequent withdrawal can cause psychiatric symptoms, among which anxiety, psychosis, and depression prevail, while it also induces cognitive impairments [17–21].

Although a link has been said to exist between EtOH consumption earlier in life and later development of cocaine addiction, no studies have examined the behavioral outcomes of this pattern of drug exposure in animals. Therefore, the aim of the present research was to assess the effect of adolescent exposure to EtOH on different models of psychiatric symptoms in adult mice after chronic cocaine intake. For this purpose, three different studies were carried out with special emphasis on the possible long-term behavioral consequences of binge drinking of EtOH during adolescence after cocaine withdrawal in adulthood. In the first study, we pretreated adolescent mice chronically with EtOH following a bingeing pattern and thereafter submitted them to a chronic cocaine treatment during adulthood. In the second study, we evaluated the short-term consequences of adolescent EtOH binge drinking. In the third study, mice were exposed to the same treatment regimens as in the first study but in this case both (EtOH and cocaine) were given at the adult age. Behavioral testing began the day after drug cessation. We assessed the effect of our treatments on locomotion, anxiety-, psychotic- and depressive-like symptoms, and short-term memory in the following paradigms: open field, elevated plus maze, prepulse inhibition, tail suspension test, and object recognition, respectively. Our main hypothesis was that adolescent but not adult EtOH binge drinking will modulate the behavioral consequences of cocaine withdrawal in adulthood. The results derived from the present research highlight the increased risk of developing many behavioral alterations among subjects with a history of EtOH abuse during adolescence and cocaine consumption in the adult age.

## 2. Materials and methods

### 2.1. Animals

Taking into account the fact that epidemiological studies have shown that more than 80% of all cocaine addicts are men [2], for the present investigation we decided to use male mice (60 adolescent and 40 adult, 21 and 55 days old respectively) of the OF1 strain, purchased from Charles River (Barcelona, Spain). For each separate study an n = 10 was formed for each drug treatment group (see below), and the same animals performed all the behavioral tests for each of the studies. The subjects were all housed under standard conditions in groups of four (cage size 60 × 33 × 3 cm), at a constant temperature (21±2°C) and relative humidity (60%), with a reversed light schedule (white lights on 19:30–07:30 h). Before the beginning of the experiments, animals remained undisturbed in the colony room for an acclimatization period of 13 days. Food and water were provided *ad libitum* throughout the study (except during the behavioral tests). All the procedures involving the mice and their care complied with national, regional, and local laws and regulations, which are in accordance with Directive 2010/63/EU of the European Parliament and the Council of 22 September 2010 on the protection of animals used for scientific purposes. The protocol was approved by the Ethical Committee for Animal Experiments of the Universitat de València.

### 2.2. Drugs

All drugs were diluted in physiological saline (NaCl 0.9% w/v; Sal) and injected intraperitoneally (IP). Absolute ethanol (EtOH) with a purity of 99.99%, obtained from Scharlab S.L. (Barcelona, Spain), was dissolved to 20% (v/v) in Sal and administered at a dose of 1.25 g/kg. This dose of EtOH was based on previously published reports, showing that it is able to induce the same blood concentration of approximately 90–130 mg/dl 5–30 minutes after its administration, respectively, in both adolescent and adult OF1 mice [22]. These blood EtOH levels correspond to 33–48 g of ethanol in humans, which represents three to five alcoholic drinks, as occurs in a binge-drinking episode [2, 23]. Cocaine hydrochloride (Coca), supplied by Laboratorios Alcaliber S.A. (Madrid, Spain), was administered at doses of 5, 15 and 25 mg/kg.

### 2.3. General behavioral procedures

#### 2.3.1. Open field

The open field (OF) experiment was performed with the aim of assessing whether our pharmacological manipulations induced any unspecific effect on the normal spontaneous locomotor activity of mice that could mask the results obtained in the rest of the experiments. For that purpose, mice were introduced individually into locomotor activity chambers consisting of a square arena (30 × 30 × 35 cm) illuminated by a dim white light (40 lx). Horizontal locomotion was recorded by a computerized video-tracking system as cm traveled in 10 min. The movement of the mice inside the open-field chamber was registered and sent automatically to the computer using the software package Ethovision 2.0. (Noldus, The Netherlands).

#### 2.3.2. Elevated plus maze

It is well established that discontinuation of cocaine after its chronic administration can cause anxiety-like symptoms in rodents [18, 24]. Thus, the present experiment evaluated the effect of our pharmacological manipulations on anxiety using the elevated plus maze (EPM) paradigm. This test is based on the natural aversion of mice to open elevated areas, and also on the natural spontaneous exploratory behavior they exhibit in novel environments. It thus measures the extent to which the rodents avoid high open spaces. The EPM consisted of two open arms (30 × 5 cm) and two enclosed arms (30 × 5 cm), and the junction of the four arms formed a central platform (5 × 5 cm). The floor of the maze was made of black Plexiglas and the walls of the enclosed arms were made of clear Plexiglas. The open arms had a small edge (0.25 cm) to provide the animals with additional grip. The entire apparatus was elevated 45 cm above floor level. The total time spent in the open and closed arms, the number of entries into the open and closed arms, and the percentage of time and entries in the open arms are commonly considered indicators of open space-induced anxiety in mice. So, the higher the number and percentage of entries and time spent in the open arms, and the shorter the time and the lower the number of entries performed in the closed arms, the lower anxiety levels are considered to be, and vice versa. Moreover, the total entries in the closed arms are regarded as locomotor activity scores [25–26].

In order to facilitate adaptation, mice were transported to the dimly illuminated test room 1 h prior to testing. At the beginning of each trial, subjects were placed on the central platform so that they were facing an open arm and were allowed to explore it for 5 min. The maze was cleaned thoroughly with a damp cloth after each trial. The behavior displayed by the mice was video recorded and later analyzed by a ‘blind’ observer using a computerized method. The following measurements were taken into account for the statistical analyses: percentage of time in the OA (% time OA), percentage of entries into the OA (% entries OA) and total entries into the closed arms (CA entries).

#### 2.3.3. Prepulse inhibition

Prepulse inhibition (PPI) of the acoustic startle response has been widely used as a measure of sensorimotor gating [27–28]. It usually occurs when a low acoustic pre-stimulus of varying intensity (20 ms duration; 74–90 dB) is given 100 ms prior to a louder startle stimulus (e.g., a pulse of 40 ms duration, 120 dB), diminishing the response to the louder stimulus. This paradigm has been used to explore the information-processing deficits that typically occur in subjects with psychosis, because human patients suffering from schizophrenia have PPI impairments: that is, they exhibit the startle response even when the pulse is preceded by a weak stimulus [29–30]. This is a cross-species translational model that allows some features of schizophrenia to be studied [27, 31–32]. Therefore, given that it has been demonstrated that cocaine withdrawal is often accompanied by psychotic symptoms [20], in the present experiment we evaluated the effect of our treatments on the PPI response. To this end, we used a PPI apparatus consisting of a Plexiglas tube (28 × 15 × 17 cm) with a platform containing a sensor on its base, so that if the animal moves, the force exerted on the platform is detected. The maximum value of this transition strength is the measure of the amplitude of the startle response. The movements caused by the startle are transduced by an accelerometer and the signal is recorded and digitized by a microcomputer that is also used to present the stimulus and collect the data. The unit is located in a soundproofed chamber (90 × 55 × 60 cm) that is constantly lit (lamp 10 w) and equipped with a loudspeaker located in the interior of the roof of the box that produces a constant sound as background noise. Two 28-cm speakers located at 15 cm from the two sides of the Plexiglas box produce acoustic stimuli. These speakers are connected to an amplifier, which in turn is connected to a noise generator that manages the startle stimulus and a second noise generator that produces the signal corresponding to the prepulse. The apparatus (mod startle response CERS) and program that collect the data were purchased from CIBERTEC, S.A. (Madrid, Spain).

Based on the methodology described by Valsamis and Schmid [33], the procedure was carried out in three phases. The first was the acclimatization phase, in which mice were placed in the animal holder for 5 minutes with a background noise of 65 dB but no startle stimuli. The following day, the second phase began, known as the acclimatization + input/output phase. This phase allowed us to establish the base level of the startle response that we would use as the main stimulus. For that purpose, we firstly implemented an acclimatization period of 5 minutes with a constant background white noise of 65 dB. Subsequently, the startle stimulus (20 ms white noise) was emitted every 20 sec, starting at 70 dB. The intensity of the startle stimulus was increased by 5 dB between each trial until it reached 120 dB, resulting in a total of 11 startle stimuli assays. Then, the third phase (acclimatization + block prepulse inhibition) was initiated, in which mice were firstly placed into the animal holder for 5 minutes as described above. The block prepulse inhibition was then started in order to measure PPI. In this assay, trials with a startle pulse alone and trials with a prepulse were pseudo-randomized. Background noise was 65 dB and the startle stimulus was 120 dB. We used two different prepulse intensities (75 and 85 dB) of 4 ms each. The 75–85 dB prepulses were also presented as single 20 ms pulses to verify that they were not acting as full pulses and did not distort the startle reaction induced by the 120 dB stimulus. As a result, there were two different prepulse trials, plus the startle pulse alone trials, plus two single prepulse trials that were pseudo-randomized and displayed 10 times each, with a total of 50 trials.

Once it had been verified that the prepulses of 75 and 85 dB did not act as startling pulses on their own and that the 120 dB stimulus elicited the higher startle reaction in all the assays, the data taken into account for the statistical analyses were those derived from the trials in which a prepulse of 75 or 85 dB was delivered 30 ms prior to the 120 dB startle pulse. PPI was calculated as a percentage score for each prepulse trial type: PPI (%) = (1-[startle response for pulse with prepulse)/(startle response for pulse alone)])×100.

#### 2.3.4. Tail suspension

The Tail Suspension Test (TST) measures the behavioral variable of immobility, which is considered to represent despair [34]. It is based on the observation that rodents, after initial escape-oriented movements, develop an immobile posture when placed in an inescapable stressful situation. In the case of the TST, the stressful situation involves the hemodynamic stress of being hung in an uncontrollable fashion by their tail [35]. This has been used as a measure of behavioral depression because, when antidepressant treatments are given prior to the test, the subjects will engage in escape-directed behaviors for longer periods of time than after treatment with the vehicle [34]. Given that cocaine withdrawal is accompanied by depression-like symptoms [36], we have investigated whether our treatment modifies the length of time spent in immobile positions in the TST. Following the protocol described by Vaugeois and colleagues [37], mice were suspended by the tail, using adhesive tape, from a hook connected to a strain gauge that recorded their movements during a 6-min test period. The behavior displayed by the mice was video recorded and later analyzed by a ‘blind’ observer using a computerized method. The parameter considered for the statistical analyses was the total time spent immobile.

#### 2.3.5. Object recognition

To estimate the effect of our treatments on memory we employed the object recognition (OR) paradigm. The novel object recognition tests episodic memory in rodents, is similar to methods used in clinical neuropsychology, and is considered a suitable model to investigate the effects of pharmacological manipulation on learning and memory [38]. It has been used in rodents as a measure of cognitive dysfunction according to deficits in object-context identification [39]. In the present study, the OR was performed in an open box (24 × 24 × 15 cm) divided by cardboard into four equally sized arenas. A camera recorder fixed to the ceiling enabled the four arenas to be visualized simultaneously. For the test, two types of objects were used: two small river stones and a small non-toxic plastic toy. This task consists of three phases: habituation, training session (T1), and test session (T2). In the habituation phase, mice were placed in the center of the empty open field box and allowed to explore it freely for 2 min. Twenty-four hours later, in T1, subjects were placed for 3 min in the open-field arena that contained the stones placed one by one in the inner and opposite corners, after which the mice were returned to their home cage for 1 min (memory retention interval). For T2, we replaced one of the stones with a toy and, after the memory retention interval, the animals were placed once again in the open-field arena for another 3 min to evaluate their exploration of the novel object. Object exploration was defined as intentional contact of the mouse's snout or front paws with the object from a distance of 2 cm or less. The following behaviors were scored: sum of the total time (t, seconds) exploring the two stones in T1, sum of the time exploring the stone and the toy in T2, and difference between the time exploring the stone and the toy in T2. The basic measure of memory acquisition in the object recognition test was the discrimination index (DI), calculated as: [DI = (tnovel—tfamiliar)/(tnovel + tfamiliar) × 100%] [40].

### 2.4. Study 1: Long-term effects of adolescent EtOH binge drinking after cocaine withdrawal in adulthood on locomotor activity, elevated plus maze, prepulse inhibition, tail suspension, and object recognition

The first study attempted to assess the long-term consequences of previous exposure to EtOH binge drinking during adolescence in adult mice withdrawn from cocaine after its chronic administration on the different behavioral paradigms described above. Thus, on postnatal day (PND) 34, corresponding to early adolescence in humans [41], animals received EtOH (or Sal in control subjects) administered 16 times in two daily injections separated by a 4-h interval on two consecutive days followed by two “drug-free” days, over a two-week period. Hence, mice received two injections on PND 34, 35, 38, 39, 42, 43, 46 and 47. This pattern of EtOH administration in rodents has been used to model the alcohol binge drinking typically observed in adolescent humans, and has been shown to induce long-term behavioral and biochemical alterations in mice [7, 23, 42]. After this treatment regime, animals were left undisturbed for three weeks, until cocaine treatment began on PND 68, an age considered to represent the beginning of adulthood in mice [43]. The procedure of cocaine administration was a variation of that described by Black and colleagues, which has been proven to induce long-lasting alterations in rodent behavior [44–45]. In short, cocaine (or Sal administered at the same volume to control individuals) was given to mice in ascending doses (binge pattern) over a period of 12 days starting on PND 68, when they received three injections per day, separated by a 60-min interval, of either Sal or 5 mg/kg of cocaine (Coca 5) on PND 68 and 69, 15 mg/kg (Coca 15) on PND 70, 71 and 72 (followed by 2-day period of abstinence), and 25 mg/kg (Coca 25) on PND 75, 76, 77, 78 and 79. As a result, the following experimental groups were constituted: Sal-Sal: the control group, which received Sal during all the experimental phases; Sal-Coca: this group received Sal during the first phase (adolescence) and cocaine during the second (adulthood); EtOH-Sal: this group was treated with EtOH in adolescence and with Sal in adulthood; EtOH-Coca: this group was injected with EtOH during adolescence and with cocaine during adulthood.

The behavioral testing began 24 h after the last injection, and was performed on subsequent days as follows: locomotor activity and elevated plus maze (PND 80), prepulse inhibition (PND 81–82), tail suspension (PND 84), and object recognition (PND 83–84). These time intervals between cocaine cessation and behavioral testing were based on previously published reports, which showed that the effects of cocaine abstinence on different behaviors in mice can be detected between at least the first and the fourteenth days after cocaine removal [18, 46].

### 2.5. Study 2: Short-term effects of adolescent EtOH binge drinking on locomotor activity, elevated plus maze, prepulse inhibition, tail suspension, and object recognition

Given that in Study 1 we found that our EtOH binge-drinking procedure administered during adolescence modulated several behaviors when measured in adulthood, we assessed here the short-term effects of this treatment under our experimental conditions. For this purpose, adolescent mice were treated with Sal or EtOH following the same schedule as in Study 1 (i.e., from PND 34 to 47), and 24 h after the last injection the experiments were conducted as follows: locomotor activity and elevated plus maze (PND 48), prepulse inhibition (PND 49–50), tail suspension (PND 52), and object recognition (PND 51–52).

### 2.6. Study 3: Long-term effects of previous EtOH binge drinking after later cocaine withdrawal in adulthood on locomotor activity, elevated plus maze, prepulse inhibition, tail suspension, and object recognition

In order to further investigate whether adolescence rather than adulthood is a critical stage for the long-term deleterious effects of EtOH on the behavioral alterations evoked by chronic cocaine intake, in the present study both treatments were given during adulthood. Therefore, here we evaluated the long-term consequences of the previous administration of EtOH binge drinking on posterior cocaine withdrawal in adult mice, on the aforementioned behavioral paradigms. In this study we used adult mice that arrived at the animalarium on PND 55, and after the 13 days' acclimatization period they were given the EtOH treatment following the same protocol as in Study 1. Hence, mice received two injections of Sal or EtOH, separated by a 4-h interval, on PND 68, 69, 72, 73, 76, 77, 80 and 81. Three weeks thereafter, on PND 102, they were treated with the cocaine regime described above, that is, they received Sal or Coca 5 on PND 102 and 103, Coca 15 on PND 104, 105 and 106, and Coca 25 on PND 109, 110, 111, 112, and 113, resulting in the same experimental design as in Study 1.

Twenty-four hours after the last treatment, the behavioral experiments started in the same sequence as in the previous study: locomotor activity and elevated plus maze (PND 114), prepulse inhibition (PND 115–116), tail suspension (PND 118), and object recognition (PND 117–118).

An illustrative summary of the experimental procedures followed in the different studies is provided in Table 1.

**Table 1:** Summary of the experimental design. EtOH = ethanol (1.25 g/kg) administered 16 times in two daily intraperitoneal (i.p.) injections separated by a 4-h interval on two consecutive days followed by a 48-h “drug-free” period over 14 days. Coca = cocaine given for three daily i.p. injections separated by a 60-min interval over a period of 12 subsequent days in ascending doses as follows: two days of 5 mg/kg, three days of 15 mg/kg, two-day period of abstinence, and five days of 25 mg/kg. Sal = saline (NaCl 0.9% w/v) given at the same treatment conditions as EtOH and coca depending on the group (control mice). OF = open field, EPM = elevated plus maze, PPI = prepulse inhibition, OR = object recognition, TST = tail suspension test. PND = Post-natal day. The experimental groups constituted were: Sal-Sal, Sal-Coca, EtOH-Sal, EtOH-Coca (in Study 1 and 3), Sal, and EtOH (in Study 2). For more details see the materials and methods section.

		TREATMENTS		BEHAVIORAL TESTING			
		SAL/ETOH	SAL/COCA	OFEPM	PPI	OR	ORTST
STUDY 1	PND	34–47	68–79	80	81–82	83	84
STUDY 2	PND	34–47	-	48	49–50	51	52
STUDY 3	PND	68–81	102–113	114	115–116	117	118

### 2.7. Statistical analyses

For Study 1 and 3, data were analyzed by means of two-way ANOVAs with two between-subjects variables: *Pretreatment* (with two levels: Sal and EtOH) and *Treatment* (with two levels: Sal and Coca). Tukey's honest significant difference (HSD) post hoc tests were carried out to evaluate the differences between experimental subgroups because this test does not require a significant interaction between factors and it is highly conservative against type I error [47]. This type of statistical analysis has previously been used by other authors following similar behavioral paradigms [48–50]. In Study 2 data were analyzed by a Student's T test. The alpha level was set at p<0.05 for all analyses. The statistical computer program Statistica 7.0 (Statsoft Inc., Tulsa, OK, USA) was employed in this investigation.

## 3. Results

**Fig 1:**
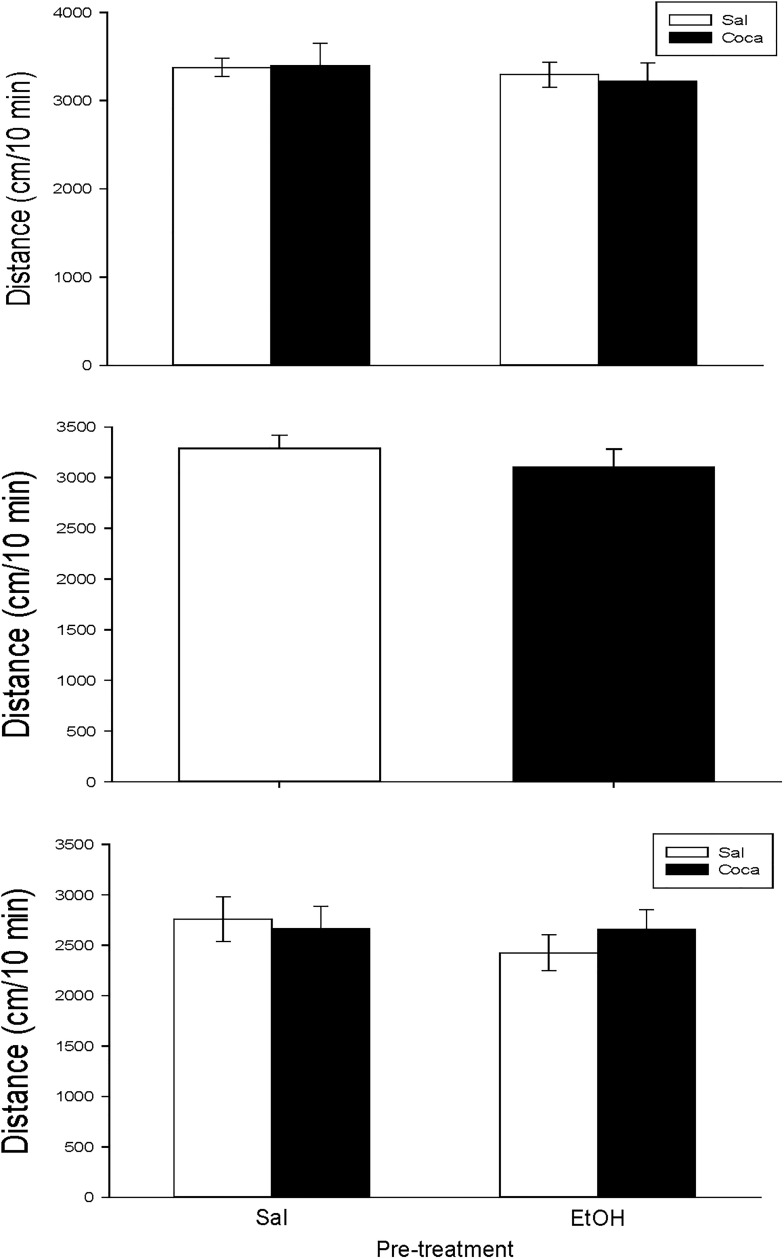
**a, b and, c. Effects of the different drug treatment conditions on locomotion.** Long-term effects of adolescent EtOH binge drinking after cocaine withdrawal in adulthood on locomotor activiy (PND 80, panel a); short-term effects of adolescent EtOH binge drinking on locomotor activity (PND 48, panel b); long-term effects of previous EtOH binge drinking after later cocaine withdrawal in adulthood on locomotor activity (PND 114, panel c). Bars depict mean ± SEM of the horizontal locomotion travelled in 10 min.

None of our treatments had an effect on the locomotor activity of mice in any of the studies (Fig 1).

### 3.1. Study 1: Long-term effects of adolescent EtOH binge drinking after cocaine withdrawal in adulthood on elevated plus maze, prepulse inhibition, tail suspension, and object recognition

Table 2 presents the results obtained from the different variables measured in the EPM for Study 1. In the case of the variable % time OA there is a significant effect for Treatment [*F*(1, 36) = 5.45, *p*<0.05], and Interaction [*F*(1, 36) = 13.63, *p*<0.01].The post hoc test showed that the % time OA spent by the Sal-Coca group was significantly lower than the Sal-Sal group (*p*<0.01). Regarding the % entries OA, we found a significant effect for both Pretreatment [*F*(1, 36) = 7.89, *p*<0.01], Treatment [*F*(1, 36) = 11.82, *p*<0.01], and Interaction [*F*(1, 36) = 5.53, *p*<0.05]. Post hoc analyses demonstrated that the % entries OA was lower in the Sal-Coca group with respect to the rest of the mice (*p*<0.01). For CA entries, the ANOVA reflected an effect of Pre-treatment and Treatment [*F*(1, 36) = 6.40, *p*<0.05] and [*F*(1, 36) = 7.28, *p*<0.05], respectively. The Tukey HSD test indicated that the Sal-Coca group performed more CA entries than the rest of animals (*p*<0.05).

**Table 2:** Long-term effects of adolescent EtOH binge drinking after cocaine withdrawal in adulthood on elevated plus maze: PND 80. Over PND 34–47 (adolescence) mice (*n* = 10 per group) were pretreated with Sal or EtOH (1.25 g/kg administered 16 times in two daily i.p. injections separated by a 4-h interval on two consecutive days followed by a 48-h “drug-free” period). In adulthood (21 days later) they were treated with Sal or Coca in three daily i.p. injections separated by a 60-min interval according to the following regime: 5 mg/kg on PND 68 and 69, 15 mg/kg from PND 70 to 72, a 2-day abstinence period, and 25 mg/kg from PND 75 to 79 (** *p*<0.01 significantly different from its respective Sal-Sal control group; # and ## *p*<0.05 and 0.01 respectively, significantly different from the rest of the groups for each corresponding variable).

	Sal-Sal	Sal-Coca	ETOH-Sal	ETOH-Coca
**% time OA**	42±6.5	11±2.5**	28±5.4	35±4.9
**% entries OA**	47±6.6	16±4##	49±6	43±4.3
**CA entries**	23±3.4	36±2.1#	20±2.6	24±3.5

**Fig 2:**
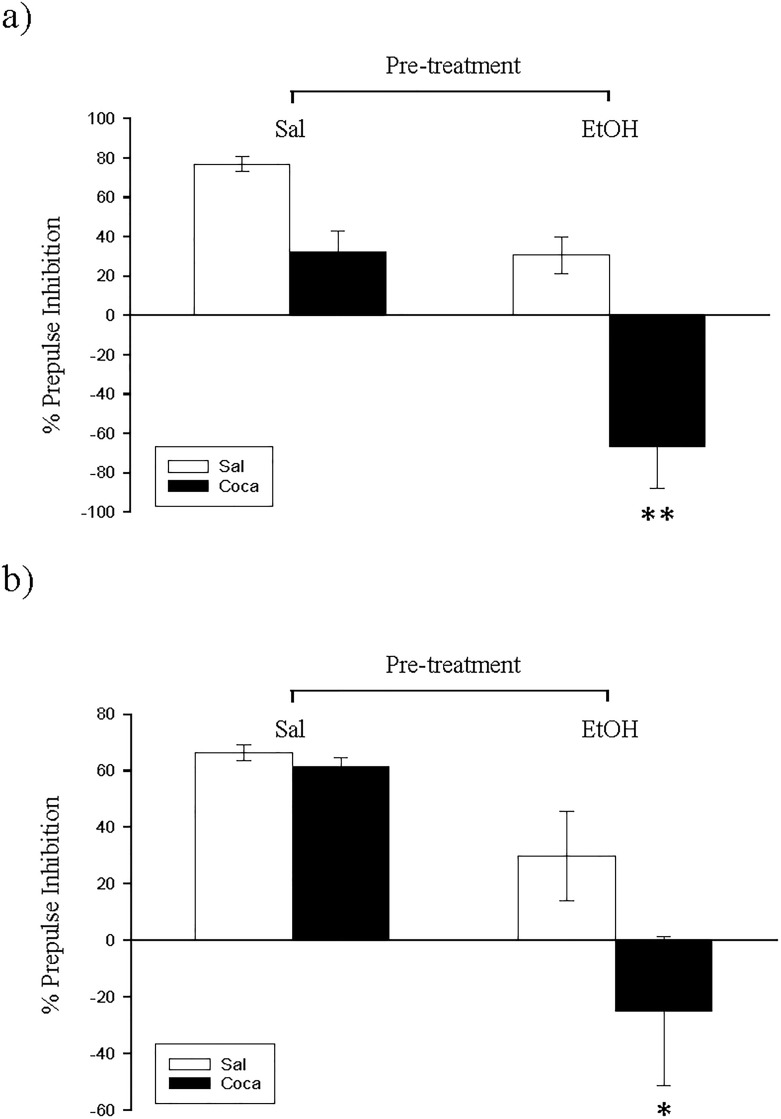
**a and b. Long-term effects of adolescent EtOH binge drinking after cocaine withdrawal in adulthood on prepulse inhibition (PND 81–82).** During adolescence mice (*n* = 10 per group) were pretreated for 14 days (PND 34–47) with Sal or EtOH (1.25 g/kg administered 16 times in two daily i.p. injections separated by a 4-h interval on two consecutive days followed by a 48-h “drug-free” period), and in adulthood with Sal or Coca by three daily i.p. injections separated by a 60-min interval according to the following regime: 5 mg/kg on PND 68 and 69, 15 mg/kg from PND 70 to 72, a 2-day abstinence period, and 25 mg/kg from PND 75 to 79. Animals were tested on PND 82. Bars depict mean ± SEM of percentage of the PPI response for trials with prepulses of 75 dB (panel a) and 85 dB (panel b) for all groups (** and * *p*<0.01 and 0.05 respectively, significantly different from the rest).

The results of the PPI experiment are displayed in Fig 2A and 2B). The ANOVA of the data obtained with a 75 dB prepulse trials (Fig 2A) revealed a significant effect of Pretreatment [*F*(1, 36) = 30.52, *p*<0.01], Treatment [*F*(1, 36) = 30.02, *p*<0.01], and Interaction [*F*(1, 36) = 4.27, *p<*0.05]. Pairwise comparisons demonstrated that the PPI of the EtOH-Coca group was significantly lower than the rest (*p*<0.01). The ANOVA of the data obtained with 85-dB prepulse trials (Fig 2B) showed a significant effect for Pretreatment [*F*(1, 36) = 4.32, *p*<0.01] and Treatment [*F*(1, 36) = 3.8, *p*<0.05]. Post hoc analyses indicated that the PPI of the EtOH-Coca group was significantly diminished with respect to the other groups (*p*<0.05).

**Fig 3:**
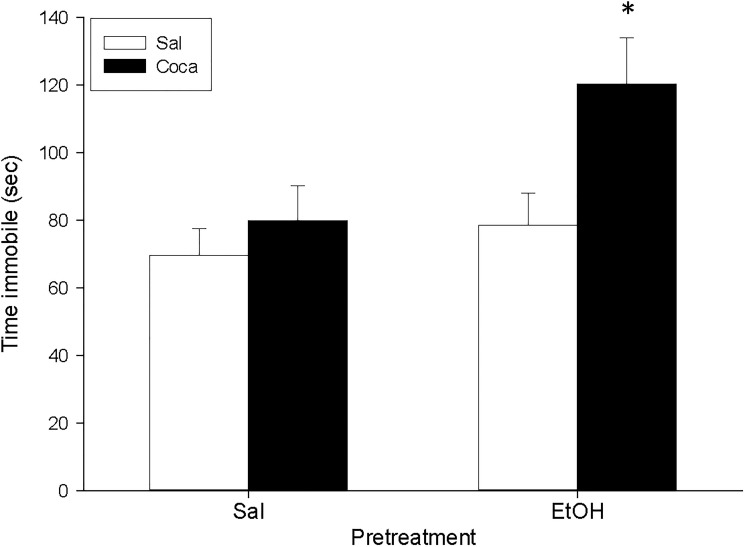
Long-term effects of adolescent EtOH binge drinking after cocaine withdrawal in adulthood on tail suspension (PND 84). During adolescence mice (*n* = 10 per group) were pretreated for 14 days (PND 34–47) with Sal or EtOH (1.25 g/kg administered 16 times in two daily i.p. injections separated by a 4-h interval on two consecutive days followed by a 48-h “drug-free” period), and in adulthood with Sal or Coca by three daily i.p. injections separated by a 60-min interval according to the following regime: 5 mg/kg on PND 68 and 69, 15 mg/kg from PND 70 to 72, a 2-day abstinence period, and 25 mg/kg from PND 75 to 79. Animals were tested on PND 84. Bars depict mean ± SEM of the time (seconds) during which the mice remained immobile in the tail suspension test (* *p*<0.05 significantly different from the rest).

For the TST (Fig 3), the ANOVA indicated a significant effect of Pretreatment [*F*(1, 36) = 5.49, *p*<0.05] and Treatment [*F*(1, 36) = 6.2, *p*<0.05]. A Tukey HSD post hoc test indicated that the time spent immobile was higher in the EtOH-Coca group in comparison to the rest of the groups (*p*<0.05).

**Fig 4:**
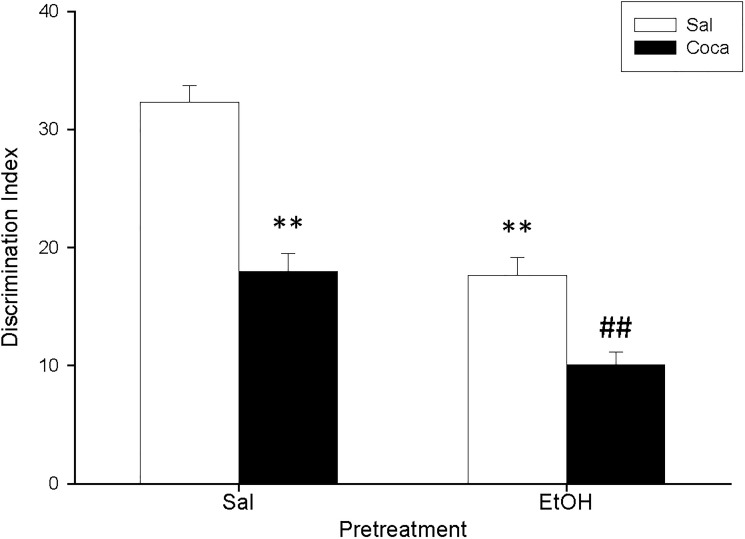
Long-term effects of adolescent EtOH binge drinking after cocaine withdrawal in adulthood on object recognition (PND 83–84). During adolescence mice (*n* = 10 per group) were pretreated for 14 days (PND 34–47) with Sal or EtOH (1.25 g/kg administered 16 times in two daily i.p. injections separated by a 4-h interval on two consecutive days followed by a 48-h “drug-free” period), and on adulthood with Sal or Coca by three daily i.p. injections separated by a 60-min interval according to the following regime: 5 mg/kg on PND 68 and 69, 15 mg/kg from PND 70 to 72, a 2-day abstinence period, and 25 mg/kg from PND 75 to 79. Animals were tested on PND 84. Bars depict mean ± SEM of the DI for all groups (** *p*<0.01 significantly different from the Sal-Sal group; ## *p*<0.01 significantly different from the rest).

The ANOVA of the data for the OR (Fig 4) indicated a significant effect of Pretreatment [*F*(1, 36) = 60.06, *p*<0.01], Treatment [*F*(1, 36) = 59.93, *p*<0.01], and also Interaction [*F*(1, 36) = 5.82, *p<*0.05]. Post hoc analyses displayed that, in comparison with the Sal-Sal group, administration of any of the pharmacological manipulations (EtOH and Coca alone or in combination) reduced the DI of the mice (*p*<0.01). Moreover, the DI of the EtOH-Coca group was significantly lower than that of the rest of the groups (*p*<0.01).

### 3.2. Study 2: Short-term effects of adolescent EtOH binge drinking on elevated plus maze, prepulse inhibition, tail suspension, and object recognition

Table 3 presents the results obtained from the different variables measured in the EPM for Study 2. The Student's T test revealed that the % time OA [*F*(1, 18) = 5.87, *p*<0.05,] and % entries OA [*F*(1, 18) = 4.3, *p*<0.05] were lower in the EtOH group. Moreover, the CA entries [*F*(1, 18) = 5.69, *p*<0.05] were higher in this group compared to the Sal.

**Table 3:** Short-term effects of adolescent EtOH binge drinking on elevated plus maze: PND 48. Across PND 34–47 (adolescence) mice (*n* = 10 per group) were pretreated with Sal or EtOH (1.25 g/kg administered for 16 times in 2 daily i.p. injections separated by a 4-h interval on 2 consecutive days followed by a 48 h “drug-free” period), and 24 h after (PND 48) they were tested (* *p*< 0.05 significantly different from its respective Sal control group).

	Sal	ETOH
**% time OA**	18±2.5	10±2.5*
**% entries OA**	42±3.8	29±5.2*
**CA entries**	38±6.6	61±6.5*

**Fig 5:**
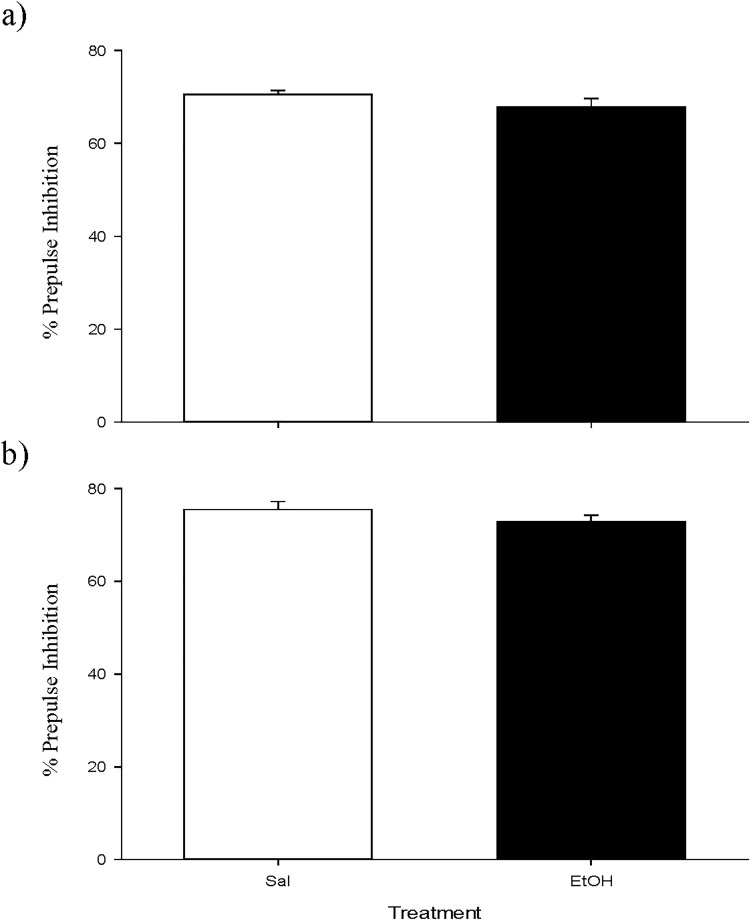
**a and b. Short-term effects of adolescent EtOH binge drinking on prepulse inhibition (PND 49–50).** During adolescence mice (*n* = 10 per group) were pretreated for 14 days (PND 34–47) with Sal or EtOH (1.25 g/kg administered 16 times in two daily i.p. injections separated by a 4-h interval on two consecutive days followed by a 48-h “drug-free” period). Animals were tested on PND 50. Bars depict mean ± SEM of percentage of the PPI response for trials with prepulses of 75 dB (panel a) and 85 dB (panel b) for all groups.

Regarding the PPI experiment, no differences were encountered between groups (Fig 5).

**Fig 6:**
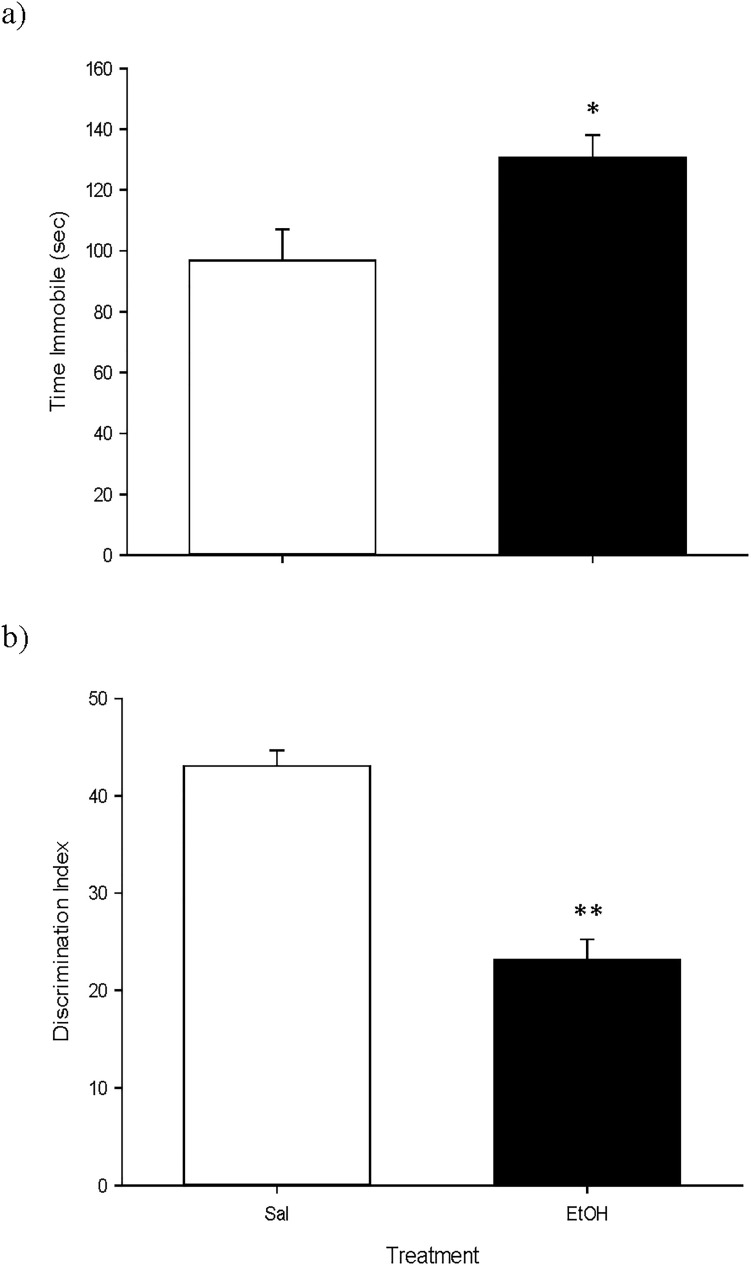
**a and b. Short-term effects of adolescent EtOH binge drinking on tail suspension (PND 52, panel a) and object recognition (PND 51–52, panel b).** During adolescence mice (*n* = 10 per group) were pretreated for 14 days (PND 34–47) with Sal or EtOH (1.25 g/kg administered 16 times in two daily i.p. injections separated by a 4-h interval on two consecutive days followed by a 48-h “drug-free” period). Animals were tested on PND 52. Bars depict mean ± SEM of the time (seconds) during which the mice remained immobile in the tail suspension test (Fig 4A); and the DI for object recognition (Fig 4B) (* and ** *p*<0.05 and 0.01 respectively, significantly different from the Sal group).

The results for the TST experiment (Fig 6A) indicated that the time spent immobile was higher in mice previously treated with EtOH [*F*(1, 18) = 7.03, *p*<0.05]; and for the OR experiment (Fig 6B) we found that the DI of the EtOH group was significantly lower than that of the Sal group [*F*(1, 18) = 57.44, *p*<0.01].

### 3.3. Study 3: Long-term effects of previous EtOH binge drinking after later cocaine withdrawal in adulthood on elevated plus maze, prepulse inhibition, tail suspension, and object recognition

**Fig 7:**
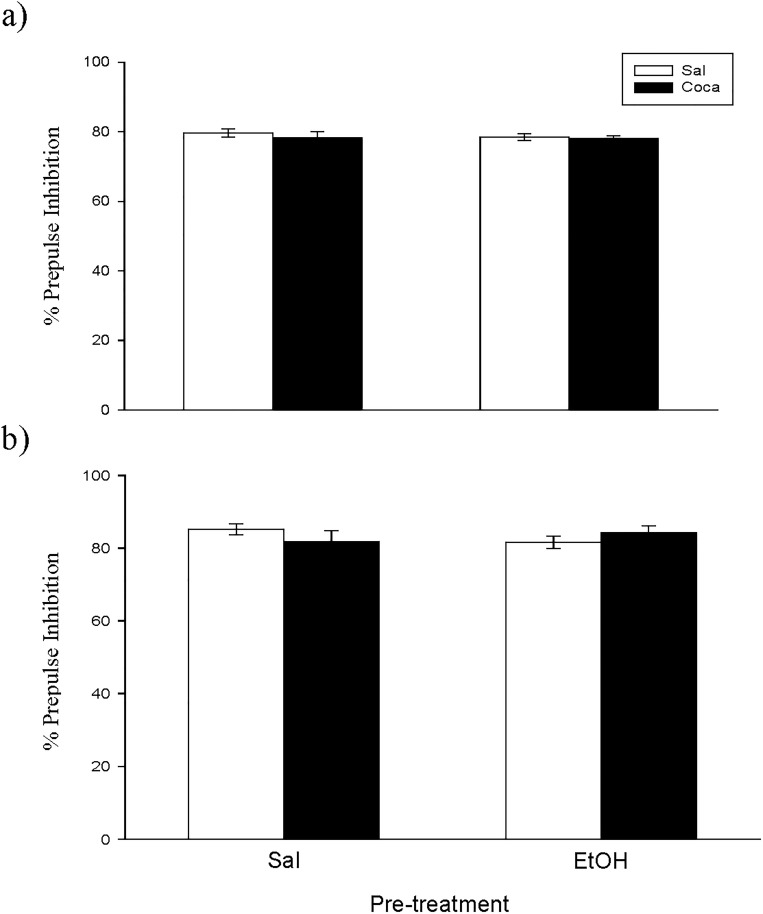
**a and b. Long-term effects of previous EtOH binge drinking after later cocaine withdrawal in adulthood on prepulse inhibition (PND 115–116).** Over PND 68–81 mice (*n* = 10 per group) were pretreated with Sal or EtOH (1.25 g/kg administered 16 times in two daily i.p. injections separated by a 4-h interval on two consecutive days followed by a 48-h “drug-free” period). Twenty-one days later, animals were treated with Sal or Coca in three daily i.p. injections separated by a 60-min interval according to the following regime: 5 mg/kg on PND 102 and 103, 15 mg/kg from PND 104 to 106, a 2-day abstinence period, and 25 mg/kg from PND 109 to 113. Animals were tested on PND 116. Bars depict mean ± SEM of percentage of the PPI response for trials with prepulses of 75 dB (panel a) and 85 dB (panel b) for all groups.

**Fig 8:**
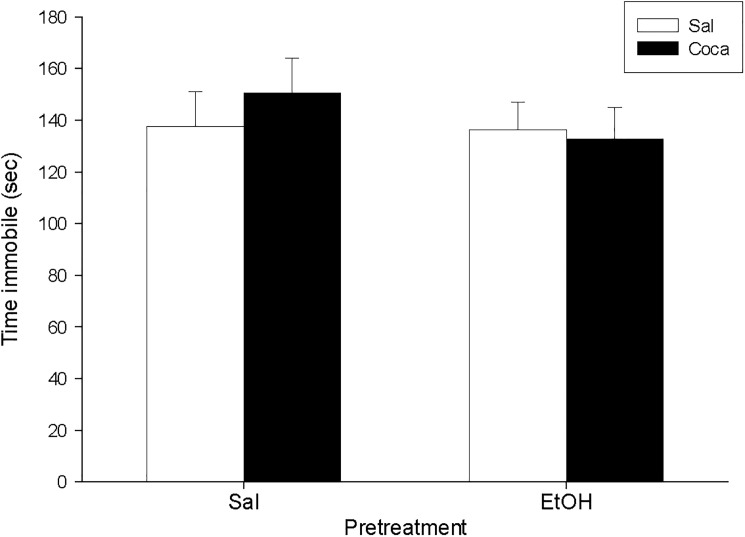
Long-term effects of previous EtOH binge drinking after later cocaine withdrawal in adulthood on tail suspension test (PND 118). Over PND 68–81 mice (*n* = 10 per group) were pretreated with Sal or EtOH (1.25 g/kg administered 16 times in two daily i.p. injections separated by a 4-h interval on two consecutive days followed by a 48-h “drug-free” period). Twenty-one days later, animals were treated with Sal or Coca in three daily i.p. injections separated by a 60-min interval according to the following regime: 5 mg/kg on PND 102 and 103, 15 mg/kg from PND 104 to 106, a 2-day abstinence period, and 25 mg/kg from PND 109 to 113. Animals were tested on PND 118. Bars depict mean ± SEM of the time (seconds) during which the mice remained immobile.

**Fig 9:**
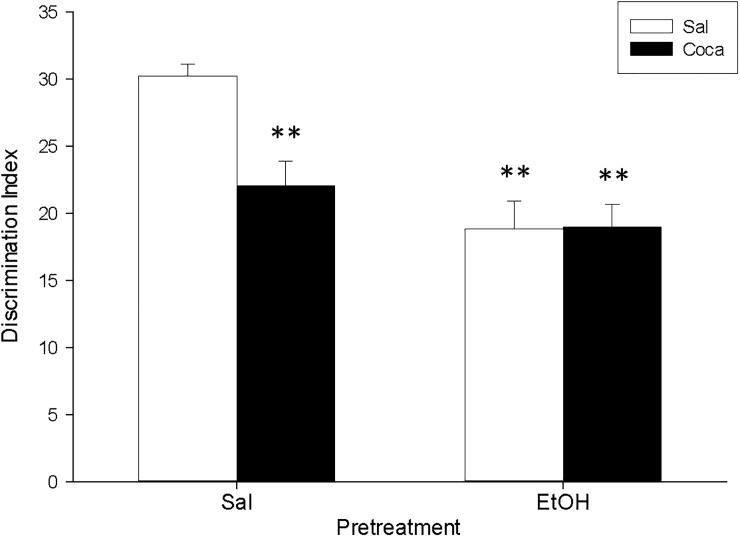
Long-term effects of previous EtOH binge drinking after later cocaine withdrawal in adulthood on object recognition (PND 118). Over PND 68–81 mice (*n* = 10 per group) were pretreated with Sal or EtOH (1.25 g/kg administered 16 times in two daily i.p. injections separated by a 4-h interval on two consecutive days followed by a 48-h “drug-free” period). Twenty-one days later, animals were treated with Sal or Coca in three daily i.p. injections separated by a 60-min interval according to the following regime: 5 mg/kg on PND 102 and 103, 15 mg/kg from PND 104 to 106, a 2-day abstinence period, and 25 mg/kg from PND 109 to 113. Animals were tested on PND 118. Bars depict mean ± SEM of the DI for all groups (** *p*<0.01 significantly different from the Sal-Sal group).

No differences were encountered between experimental subgroups in the EPM, the PPI, and the TST for Study 3 (Figs 7 and 8, and Table 4). Nonetheless, for the OR experiment (Fig 9) we found a significant effect of Pretreatment [*F*(1, 36) = 18.07, *p*<0.01], Treatment [*F*(1, 36) = 5.51, *p*<0.05], and Interaction [*F*(1, 36) = 6.03, *p*<0.05]. The post hoc analyses revealed that the DI of the EtOH-Sal, the Sal-Coca, and the EtOH-Coca groups was lower than that of the Sal-Sal group (*p*<0.01).

**Table 4:** Long-term effects of previous EtOH binge drinking after later cocaine withdrawal in adulthood on elevated plus maze: PND 114. Across PND 68–81 mice (*n* = 10 per group) were pretreated with Sal or EtOH (1.25 g/kg administered for 16 times in 2 daily i.p. injections separated by a 4-h interval on 2 consecutive days followed by a 48 h “drug-free” period). Twenty one days later, animals were treated with Sal or coca in three daily i.p. injections separated by a 60-min interval according to the ensuing regime: 5 mg/kg on PND 102 and 103, 15 mg/kg from PND 104 to 106, a 2-day abstinence period, and 25 mg/kg from PND 109 to 113.

	Sal-Sal	Sal-Coca	ETOH-Sal	ETOH-Coca
**% time OA**	32±3.4	30±3.2	31±4.5	29±3.6
**% entries OA**	52±3.6	53±5.5	48±4.2	54±5.8
**CA entries**	33±5	33±6	43±6	35±9.2

## 4. Discussion

The current data represent the first approach towards assessing the long-lasting behavioral consequences of adolescent exposure to EtOH on cocaine withdrawal in adult mice. We used a murine model of pubertal binge administration of alcohol followed by a pattern of chronic binge cocaine intake in adulthood [42, 45], thereby imitating a pattern seen in many human polydrug users [1, 8]. The main important results of the present research are derived from Study 1, where we showed that this type of drug consumption in adolescent mice increases the vulnerability to develop some behavioral alterations in adulthood, which could be related to the high incidence of mental disorders in polydrug users. Thus, by using the PPI and the TST paradigms we found that an early history of EtOH ingestion enhances the risk of suffering from psychotic- and depressive-like symptoms, respectively, after withdrawal of chronic cocaine at an adult age. Furthermore, we found that the cognitive deficits in the OR task evoked by these drugs when given separately are boosted in mice exposed to EtOH during adolescence and, thereafter, to cocaine. In addition, we show that adolescent EtOH bingeing reduces the anxiogenic-like effects observed in the EPM after cocaine abstinence in adult mice. Adolescent animals tested just after the removal of EtOH binge also displayed anxiety- and depressive-like symptoms, as well as cognitive impairments compared to control mice (Study 2), thereby confirming the higher sensitivity to the effect of EtOH in this period of development. In contrast, when these drugs were given in the adult age we found only a deterioration in the OR task and, more importantly, we did not observe any potentiation when both treatments were combined during adulthood (Study 3). Overall, the present investigation supports the idea that adolescence is a critical stage for the deleterious effects of EtOH binge drinking, and that this kind of consumption may deteriorate the behavioral consequences of later cocaine withdrawal. An illustrative summary of the findings obtained across the different studies is presented in Table 5.

**Table 5:** Simplified summary of the main results derived from the different studies.

Pre-treatment	Treatment	Experimental group	Anxiety (EPM)	Psychotic Symptoms (PPI)	Depressive symptoms (TST)	Cognitive disfunctions (OR)
Adolescent	Adult	EtOH-Sal	=	=	=	+
		Sal-Coca	+	=	=	+
		EtOH-Coca	=	+	+	+ + +
Adolescent	-	EtOH	+	=	+	+
Adult	Adult	EtOH-Sal	=	=	=	+
		Sal-Coca	=	=	=	+
		EtOH-Coca	=	=	=	+

### Behavioral effects of binge EtOH administration

Immediately after exposure to a chronic intermittent pattern of binge drinking during adolescence, mice showed an increase in anxiety and in immobility in the EPM and the TST, respectively. Cognitive deficits were also evident in the OR test. However, only cognitive deficits remained present three weeks after the last EtOH administration, either during adolescence or adulthood.

Adolescent mice treated with our binge EtOH regime displayed heightened anxiety scores in the EPM when tested immediately afterwards (Study 2, see Table 3). These data are in accordance with earlier reports also showing that adolescent binge EtOH treatment boosts anxiety-like behaviors in the EPM when measured the day after the end of alcohol administration in rodents [51–52]. However, we did not detect any long-term consequences of our EtOH treatment on the EPM in Study 1, when measured in adulthood (Table 2). This data is in agreement with previous research showing similar results after exposure of adolescent mice to the same EtOH binge-treatment regime and tested three weeks thereafter [23]. Furthermore, in Study 2 we found that when adolescent animals were treated with EtOH and tested shortly afterwards in the TST (i.e., five days after the end of the binge drinking administration) their immobility scores where augmented with regard to the control group (Fig 4A). This supports the notion that clinical and pre-clinical evidence shows the high degree of comorbidity of alcoholism and depression. In fact, it has been proposed that symptoms of depression during EtOH abstinence increase the likelihood of relapse and indicate a worse prognosis in terms of treatment outcome [53–54].

In agreement with earlier reports, we observed that our EtOH treatment during adolescence induced both short- (i.e., when measured 5 days after its administration; Study 2) and long-term (i.e., when measured 37 days after its administration; Study 1) memory impairments in the OR task (Figs 3 and 4B respectively) [6, 42, 55–57]. In addition, in Study 3 we found the same long-lasting effects when EtOH was given during adulthood (Fig 5). This indicates that previous EtOH treatment is able to induce memory deficits when given in both the adolescent and the adult periods, and that this effect is long-lasting in mice. Thus, it seems that memory is especially vulnerable to the deleterious effects of alcohol binge drinking.

Finally, in agreement with previous findings, EtOH binge drinking did not produce psychotic-like symptoms when administered during adolescence or adulthood, because the values of the PPI were not modified in the groups treated only with EtOH in any of the experiments [4].

Overall, we can conclude that EtOH binge drinking during adolescence, despite inducing a transient increase in anxiety and depressive-like symptoms during adolescence, only gave rise to long-lasting cognitive deficits that do not depend on the age at EtOH administration. Therefore, it seems that with the exception of memory disturbances, which may be detected both short- (Study 2) and long-term (Study 1 and 3) after EtOH administration and appear to be independent of the age of the animals, the anxiogenic- and depressive-like actions of binge drinking are only produced when EtOH is administered during adolescence, thus suggesting that these effects are age-dependent.

### Behavioral effects of cocaine withdrawal

Withdrawal from cocaine without a previous history of EtOH exposure induced memory disturbances in the OR independently of the age at which cocaine was administered. Conversely, after cocaine withdrawal only mice exposed to this drug between PND 68–79 (Study 1) displayed anxiety-like effects in the EPM, thereby suggesting that the anxiogenic-like effects evoked by cocaine abstinence may be age-dependent. Irrespective of age, no psychotic- or depressive-like symptoms were manifested by mice after cocaine discontinuation in the PPI and the TST respectively.

In relation to the possible cognitive dysfunctions elicited by cocaine abstinence assessed by the OR procedure, we found that mice undergoing withdrawal performed this task more poorly than Sal-Sal control subjects in both Study 1 (adolescents) and Study 3 (adults) (Figs 3 and 5 respectively). This result has also been shown by other researchers, who observed that extended exposure to cocaine deteriorates recognition memory in rodents following a similar procedure to that of the current research [58–59].

In Study 1 we demonstrated that cocaine withdrawal evoked anxiety-like behaviors in the EPM (Table 2). These results are in agreement with those of earlier studies in which it has been reported that cocaine discontinuation is accompanied by anxiety symptoms in rats [60–64]. However, in contrast to Study 1, the anxiety levels of mice under cocaine abstinence did not differ from those of the control group in Study 3 (data not shown). These discrepancies between the two studies may be due to the different age of drug exposure. In Study 1 cocaine was administered during PND 68–79, a period that corresponds to the beginning of the adult age (i.e., this age is considered as being “young adulthood or late adolescence”); in contrast, in Study 3 they were treated with cocaine from PND 102 to 113, an age that represents the height of adulthood in mice [41, 43]. It is well known that brain maturation in mammals occurs when the adult age is completely accomplished, as should be the case of the mice from Study 3. Consequently, it would be possible that these mice were less susceptible to the anxiogenic effects elicited by cocaine abstinence than those of Study 1 because they had a more developed brain. In agreement with this speculation, an earlier study carried out by Valzachi and colleagues [25] demonstrated that mice that were chronic cocaine-pretreated during adolescence exhibited a greater magnitude of cocaine withdrawal-induced anxiety-like behavior compared to adult males that received the same treatment conditions.

On the other hand, we found no effects of cocaine abstinence in the PPI procedure, irrespective of age (Studies 1 and 3), suggesting no significant psychotic-like actions of cocaine withdrawal in our experiments. This result is in the same line as previous studies showing that cocaine withdrawal did not modify the PPI of adult rats [65–67]. At present, data about the consequences of abstinence from cocaine in the PPI paradigm in rodents are scarce, and we have presented here the earliest investigation showing that cocaine-withdrawn mice did not manifest any psychotic-like symptoms, at least under our experimental conditions.

Similarly, regarding the TST results, we did not observe any significant alterations in the time that mice of any age remained immobile after cocaine abstinence, thereby indicating that drug discontinuation after its chronic intake did not induce any depressive-like effects. This is a very interesting result, because this is the first time that the consequences of cocaine withdrawal on the TST paradigm has been measured in mice. By using other behavioral paradigms that also model behavioral depression, such as the forced swim test and the sucrose preference test, different results had been reported in rodents. For example, in rats it has been shown that cocaine withdrawal is accompanied by depression-like symptoms [19, 68–69]. In contrast, other authors have found that abstinence from chronic cocaine administration did not induce any depression-like effects in mice, as in the present investigation [62]. Although methodological differences between studies may explain these divergent results, it could be possible that mice and rats have a different sensitivity to the depressive-like actions induced by cocaine withdrawal.

In summary, in relation to the possible behavioral disturbances evoked by cocaine cessation after its chronic administration during adulthood, we found that this treatment could induce anxiety-like effects depending on the age at which it is administered. Thus, it seems that young adult mice are more sensitive to the anxiogenic actions elicited by cocaine withdrawal than adult mice. No other difference was seen between the behavioral profile of younger and older abstinent mice, which exhibit similar cognitive dysfunctions in the OR but neither psychotic- nor depressive-like symptoms.

### Behavioral effects of previous binge administration of EtOH on cocaine withdrawal

The most important and innovative data obtained in the present research came from the results derived from the mice treated with the combination of EtOH and cocaine. In this way, with the exception of the EPM experiment, in which we did not detect any alterations in the anxiety scores in these groups, we found that exposure to EtOH during adolescence elicited both psychotic- and depressive-like effects, and enhanced memory impairments in the PPI, TST, and OR respectively after cocaine cessation. None of these outcomes were seen when cocaine-withdrawn mice were previously treated with EtOH at the adult age. Therefore, it seems that adolescent EtOH binge drinking could increase the vulnerability of mice to suffer from different behavioral disturbances after later cocaine withdrawal.

As can be observed in the EPM experiment in Study 1 (Table 2), we found that the anxiety values obtained when adolescent mice were pretreated with EtOH and later with cocaine did not differ from those of the control mice. This was a very surprising result considering that, in this same study, mice only exposed to cocaine displayed enhanced anxiety values (Table 2) and that in Study 2 we found that adolescent mice displayed short-term anxiogenic-like behaviors after binge administration of EtOH (Table 3). Hence, it may be expected that previous exposure to binge administration of EtOH during adolescence will potentiate the later anxiogenesis evoked after cocaine cessation in adults. Nevertheless, taking into account the present results, an alternative explanation may be considered. It could be possible that mice exposed to EtOH during adolescence and to cocaine in adulthood displayed lower anxiety levels than those only exposed to cocaine because they have previously experienced the anxiety evoked by EtOH abstinence during adolescence, thus making them more resilient to the anxiogenic effects of posterior cocaine withdrawal. This could be interpreted as meaning that juvenile exposure to EtOH alleviates the anxiety symptoms associated with later cocaine abstinence. In that sense, it is widely accepted that there exists a correlation between anxiety and cocaine addiction, and that high anxiety is one of the factors that contribute to relapse because many cocaine-abstinent addicts consume the drug again to alleviate the anxiety induced by the withdrawal syndrome [25, 70]. So, one can speculate that EtOH consumption in adolescence protects against cocaine relapse by reducing anxiety levels after its abstinence in adulthood. However, another possibility is that if previous chronic EtOH administration and its removal is able to reduce the posterior anxiogenic effects elicited by cocaine cessation, it could maintain cycles of consumption and abstinence because subjects experience the aversive consequences of the abstinence syndrome to a lesser degree. Notwithstanding, future studies must be carried out to test this hypothesis.

Another meaningful result comes from the finding that when juvenile mice were pretreated with EtOH and later with cocaine (EtOH-Coca group from Study 1), their PPI was decreased (Fig 1A and 1B). This is an appealing result, because neither binge EtOH administration nor cocaine discontinuation affected the PPI of animals when given alone. Nevertheless, the combination of EtOH during adolescence and cocaine in adulthood acts synergistically to impair the suppression of the startling reflex. Interestingly, in Study 3 we observed that when these same treatments were both dispensed at the adult age it did not affect the PPI of animals. Therefore, the interaction between EtOH and Coca treatments on the PPI impairment is age-dependent. Hence, given that it has been established that the PPI is a suitable paradigm to infer psychotic symptoms in rodents [28, 32], we propose that adolescence is a critical period in which EtOH binge drinking could facilitate the posterior development of psychotic symptoms after chronic cocaine ingestion at the beginning of adulthood.

Similarly to what occurred with the PPI experiment, in Study 1 we also observed that when animals were previously exposed to EtOH during adolescence and later withdrawn from cocaine, they remained significantly more time immobile in the TST (Fig 2). This suggests that a history of abuse of EtOH in adolescence and cocaine discontinuation after its chronic intake at an adult age could evoke some depressive-like symptoms in mice. In contrast, when both EtOH binge drinking and chronic cocaine dispensation were given in adulthood, the TST values were not altered (Study 3). Hence, it seems that adolescent exposure to EtOH bingeing followed by adult cocaine withdrawal is specifically susceptible to promote depression-like manifestations.

Finally, another very prominent result comes from the finding that we demonstrate how the combination of adolescent binge EtOH administration with later cocaine abstinence in adulthood boosted the detrimental effects that these two treatments may provoke in the OR when given separately. Thus, in Study 1 we reported that the DI of the EtOH-Coca group was not only lower than that of the control group, but also than those of the groups receiving EtOH or cocaine alone, which, as noted above, were already decreased with respect to the control group (Fig 3). This enhancement of memory disruption was not observed in the EtOH-Coca group from Study 3, in which both treatments were given at the adult age (Fig 5). This suggests that the combination of EtOH consumption during puberty and cocaine at the onset of adulthood potentiates the impairing effects of these substances on memory, thereby highlighting again the idea that heavy adolescent alcohol consumption could have a negative impact on the behavioral disturbances induced by later chronic cocaine intake.

In conclusion, the current results demonstrate for the first time the long-lasting consequences of juvenile exposure to EtOH on cocaine withdrawal in adult mice. Given that pubertal alcohol binge drinking followed by a later development of cocaine abuse is an increasingly common pattern of drug consumption in human beings, the results of the present research are particularly relevant, as they provide evidence of the augmented risk of developing several psychiatric illnesses in subjects with this pattern of drug intake. Nonetheless, this is a first behavioral approximation, and future studies will be required to establish the contribution of the different cerebral pathways and neurotransmitter systems involved in such effects, as well as to validate the hypotheses presented herein. In addition, given that the National Institute of Health has recommended that sex should be considered as a biological variable in all medical study design, and that it has been reported that there are sex-specific negative consequences of early adolescent ethanol exposure [71], it becomes essential to assess in the near future the possible differences between males and females in their response to our treatments. This will help to further our knowledge about the mechanisms involved in the psychopathologies shown by this kind of polydrug user, and to open up the way to the development of new pharmacological tools to prevent or palliate these disorders.
